# Feasibility and clinical applications of multiple breath wash-out (MBW) testing using sulphur hexafluoride in adults with bronchial asthma

**DOI:** 10.1038/s41598-020-58538-x

**Published:** 2020-01-30

**Authors:** Frederik Trinkmann, Steffi A. Lenz, Julia Schäfer, Joshua Gawlitza, Michele Schroeter, Tobias Gradinger, Ibrahim Akin, Martin Borggrefe, Thomas Ganslandt, Joachim Saur

**Affiliations:** 1 1st Department of Medicine (Cardiology, Angiology, Pulmonary and Intensive Care), University Medical Centre Mannheim, Medical Faculty Mannheim, Heidelberg University, Heidelberg, Germany; 2 Department of Biomedical Informatics of the Heinrich-Lanz-Centre, University Medical Centre Mannheim, Medical Faculty Mannheim, Heidelberg University, Heidelberg, Germany; 3 Institute of Clinical Radiology and Nuclear Medicine, University Medical Centre Mannheim, Medical Faculty Mannheim, Heidelberg University, Heidelberg, Germany; 4 DZHK (German Centre for Cardiovascular Research), partner site Mannheim, University Medical Centre Mannheim, Medical Faculty Mannheim, Heidelberg University, Heidelberg, Germany

**Keywords:** Asthma, Outcomes research

## Abstract

Ventilation heterogeneity is frequent in bronchial asthma and can be assessed using multiple breath wash-out testing (MBW). Most data is available in paediatric patients and using nitrogen as a tracer gas. We aimed to evaluate sulphur hexafluoride (SF_6_) MBW in adult asthmatics. Spirometry, whole-body plethysmography, impulse oscillometry and SF_6_-MBW were prospectively performed. MBW parameters reflecting global (lung clearance index, LCI), acinar (S_acin_) and conductive (S_cond_) ventilation heterogeneity were derived from three consecutive wash-outs. LCI was calculated for the traditional 2.5% and an earlier 5% stopping point that has the potential to reduce wash-out times. 91 asthmatics (66%) and 47 non-asthmatic controls (34%) were included in final analysis. LCI_2.5_ and LCI_5_ were higher in asthmatics (p < 0.001). Likewise, S_acin_ and S_cond_ were elevated (p < 0.001 and p < 0.01). Coefficient of variation was 3.4% for LCI_2.5_ and 3.5% for LCI_5_ in asthmatics. Forty-one asthmatic patients had normal spirometry. ROC analysis revealed an AUC of 0.906 for the differentiation from non-asthmatic controls exceeding diagnostic performance of individual and conventional parameters (AUC = 0.819, p < 0.05). SF_6_-MBW is feasible and reproducible in adult asthmatics. Ventilation heterogeneity is increased as compared to non-asthmatic controls persisting in asthmatic patients with normal spirometry. Diagnostic performance is not affected using an earlier LCI stopping point while reducing wash-out duration considerably.

## Introduction

Disease control is the primary goal of asthma therapy being linked to absence of symptoms and exacerbations^1^. Regrettably, up to half of the patients are poorly controlled^2,3^. Despite therapeutic advances, numbers remained fairly unaltered during the last decade.

The clinical and pathophysiological explanations associated with poor disease control are heterogenous. In general, more severe disease is related to more frequent exacerbations, health care contacts^3^ and symptoms^4^. Lung function is also impaired in severe disease indicated by a lower forced expiratory volume in one second (FEV_1_) and lower forced vital capacity (FVC)^3^. Both parameters represent rather central sites of obstruction. However, involvement of peripheral airways is common in the majority of asthmatic patients^5^. This holds true across the whole spectrum of severity^6^ and may be a consequence of several influencing factors. These include inflammation, wall thickening, smooth muscle hypertrophy, and mucus^7–10^. However, changes in the lung periphery are still often missed by commonly used techniques such as spirometry.

Impulse oscillometry (IOS) is an inexpensive non-invasive technique to measure airway resistance. It was shown to identify small airway obstruction^11^, the related characteristics of disease control^12,13^ and response to acute bronchodilator therapy^14^. Small airway dysfunction was shown to impair quality of life in smokers^15^. This even applies in absence of obstructive lung disease.

Other techniques aiming at an early diagnosis or phenotyping have also been investigated. Most recently, Sugawara and co-workers could identify three subtypes of cough variant asthma. They were able to demonstrate a differential therapeutic effect of inhaled corticosteroids (ICS)^16^. Eosinophilic airway inflammation is common in asthma and associated with increased corticosteroid responsiveness. Blood eosinophilia is already a widely used parameter in patients with severe disease planned for targeted antibody therapy in personalized medicine^17^. Single surrogate markers such as blood eosinophil count, immunoglobulin E, and fractional exhaled nitric oxide (FeNO) have overall moderate diagnostic accuracy^18^. However, it was recently demonstrated that FeNO predicts response to ICS in patients with non-specific respiratory symptoms^19^. These may represent a distinct phenotype or stage of asthmatic disease not detected using current diagnostic work-up. Ventilation heterogeneity is also frequent in obstructive lung disease and can be assessed using multiple breath wash-out testing (MBW). The technique was shown to be feasible in adult patients with chronic obstructive pulmonary disease (COPD)^20–22^ and pulmonary hypertension^23^. Asthmatic patients were found to have increased ventilation heterogeneity as compared to controls^24,25^ that could be improved by therapy^24,26,27^ and is worse during exacerbations^28^. Moreover, previous investigations also suggest a link to poorer asthma control^27,29^. An association of ventilation heterogeneity with airway responsiveness was seen in asthma but not in COPD^30^.

Hence, MBW has great potential to improve phenotyping as well as differential diagnostics. Nevertheless, several metrological issues have still to be overcome. Most MBW data in bronchial asthma has been acquired in paediatric patients using nitrogen (N_2_) as tracer gas. The latter potentially suffers from technical issues such as N_2_-back diffusion, measurement inaccuracies and vulnerability to leak flows^31–34^. Mass spectrometry using open wash-in MBW sulphur hexafluoride (SF_6_) is considered the gold standard^35^. However, this has been associated with high costs and effort. Introduction of a photo-magneto-acoustic multi-gas analyser allowed the direct measurement of SF_6_ concentrations with high accuracy^36^. Recently, this led to construction of a closed circuit SF_6_-MBW setup considerably facilitating application and reducing costs^37^. We therefore set out to evaluate the feasibility of SF_6_-MBW in adult patients with bronchial asthma and derive potential clinical applications.

## Methods

### Subjects

We prospectively evaluated patients with known or first diagnosis of bronchial asthma as well as non-asthmatic controls. Classification criteria are given in detail in the *online supplement*. The study protocol was approved by the Medical Ethics Committee II of the Medical Faculty Mannheim (Heidelberg University), compliant with the Declaration of Helsinki and registered at clinicaltrials.gov (NCT03820427). Written informed consent was obtained from all participants and/or the respective legal guardian prior to inclusion.

### Study protocol

#### Spirometry and whole-body plethysmography

All subjects underwent pre-bronchodilator conventional lung function testing including spirometry as well as whole-body plethysmography (MasterScreen Body) and were in stable clinical condition. Existing maintenance therapy was not withheld. Reversibility testing was then performed with doses of 40 µg ipratropium bromide and 100 µg fenoterol hydrobromide administered via soft-mist haler (Berodual Respimat, Boehringer Ingelheim Pharma GmbH & Co. KG, Germany). Indications include presence of either obstruction (FEV_1_/FVC < 70% of predicted), hyperinflation (residual volume/total lung capacity (RV/TLC) > 40%), and/or respective flow-volume-curves or flow-pressure curves. In addition to these parameters, maximum expiratory flow (MEF) at 75, 50 and 25% of FVC as well as functional residual capacity (FRC) were determined. Reference values were derived from the revised 1993 version of the European Community for Steel and Coal equations (ECSC)^38^.

#### Transfer factor for carbon monoxide (TLCO)

TLCO was determined pre-bronchodilator in single breath technique and corrected for ventilated alveolar volume (TLCO/VA). The difference was defined as ΔTLCO.

#### Multiple breath wash-out (MBW) testing

A commercially available closed-circuit system (Innocor, PulmoTrace ApS, Glamsbjerg, Denmark) was used for MBW measurements as previously described in detail^37^. The device consists of a 3-liter rebreathing bag filled with a mixture of room air and test gas (94% O_2_, 1% SF_6_ and 5% N_2_O, PulmoTrace ApS) from an on-board gas cylinder. Functional residual capacity (FRC), acinar (S_acin_) and conductive (S_cond_) ventilation heterogeneity were derived from three consecutive wash-outs using proprietary software provided by the manufacturer (software version 8.0 beta 1). LCI was calculated for the 2.5% (LCI_2.5_) and 5% (LCI_5_) stopping points, respectively. Subjects were breathing tidally and the test was stopped when end tidal SF_6_ had fallen below 1/40 of the starting concentration for three consecutive breaths. Wash-out was always performed until the later LCI_2.5_ stopping point. To show the time saving potential of an earlier LCI_5_ stopping point, wash-out times were calculated separately. Only patients with at least two technically acceptable LCI measurements based on slightly modified ATS/ERS criteria (online supplement) were included in final analysis. All subjects underwent three consecutive pre-bronchodilator MBW tests in upright position.

#### Impulse oscillometry (IOS)

IOS measurements were performed during tidal breathing (MasterScreen IOS, CareFusion 234 GmbH, Höchberg, Germany). We conducted three consecutive pre-bronchodilator measurements and derived parameters of airway resistance (frequency dependence D5–20) and reactance (resonance frequency F_res_, reactance area Ax), respectively. In patients undergoing reversibility testing according to above mentioned indications, three consecutive post-bronchodilator measurements were additionally performed.

All lung function tests were independently assessed by two experienced investigators. MBW and IOS were conducted prior to all forced lung function manoeuvres. The difference between FRC as determined by whole-body plethysmography and MBW was defined as ΔFRC_pleth-MBW_.

### Statistical analysis

Mean values are given ± standard deviation (SD) unless stated otherwise. Differences between groups were assessed by Student’s t-test for continuous variables or Chi-squared test for categorical variables. The coefficient of variation (CV) was calculated as SD/mean from two or three valid MBW measurements, respectively. Sample size calculation was based on previously published data^39^. An overall cohort of 60 subjects would provide 95% power to detect a 1.8 ± 0.4 difference in LCI with a 2:1 allocation of patients and non-asthmatic controls, respectively. An alpha error of less than 5% in two-sided testing was considered statistically significant. R Statistical Software (v3.4.2, Foundation for Statistical Computing, Vienna, Austria) was used for all data analysis^40^. A subgroup of asthma patients with normal lung function in conventional testing was prespecified and defined as having normal FEV_1_ (>80% of predicted) and no obstruction in spirometry (FEV_1_/FVC > 70%). All lung function parameters differing between asthma patients with normal lung function and non-asthmatic controls in univariate analysis (p ≤ 0.05) were included in an elastic-net generalized linear model corrected for age. L1 regularization was used for variable selection based on a k-fold cross validation approach to find optimal hyper-parameters. Diagnostic performance was evaluated using receiver operating curve (ROC) analysis and calculation of area under the curve (AUC).

## Results

Final analysis was performed in 91 patients with bronchial asthma (66%) and 47 non-asthmatic controls (34%) with valid data sets. Only two valid MBW measurements could be obtained in 11 non-asthmatic controls (23%) and 32 asthmatics (35%, p = 0.2). Five asthmatic patients (3.4%) had to be excluded due to the prespecified acceptability criteria for MBW testing whereas in another 4 patients (2.7%) only one valid MBW measurement could be obtained. Baseline characteristics are given in Table 1. Reversibility testing was performed in 63 patients (69% of asthmatic patients) with details given in Supplementary Table S1. Positive rates were 38% for FEV_1_ and Ax, 21% for RV and 29% for D5–20, respectively.

**Table 1:** Baseline characteristics.

	unit	Asthma (n = 91)		Controls (n = 47)		p-value
		value	range	value	range	
age	years	55 ± 18	17–88	47 ± 19	21–85	0.02*
height	cm	173 ± 10	152–198	170 ± 9	145–188	0.02*
weight	kg	79 ± 21	50–132	81 ± 17	45–123	0.54
mMRC		1.1 ± 1.2	0–4	0	0	<0.0001*
**smoker**						
yes/ex/never	n	18/36/37		4/12/31		0.02*
	%	20/40/40		9/25/66		

mMRC: modified Medical Research Council dyspnoea scale. *Statistically significant p < 0.05.

**Figure 1:**
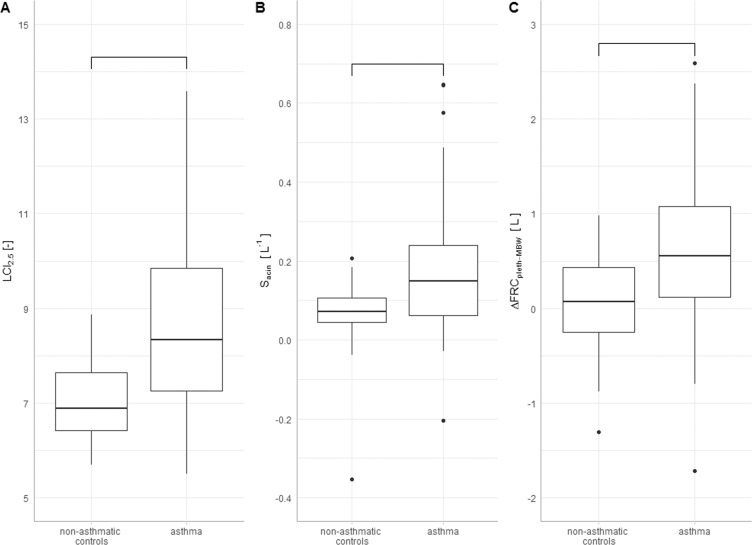
MBW parameters. Box-and-whisker-plots for (**A**) LCI_2.5_ (p < 0.001), (**B**) S_acin_ (p < 0.001) and (**C**) ΔFRC_pleth-MBW_ (p<<0.001) separated for asthma patients and non-asthmatic controls. Connectors indicate statistically significant (p < 0.05) differences.

All lung function parameters differed between asthmatic patients and non-asthmatic controls except for total lung capacity (TLC), functional residual capacity as determined by whole-body plethysmography (FRC_pleth_) and ΔTLCO (Table 2). Forty-one asthma patients had normal lung function in spirometry with details of differences given in Supplementary Table S2. LCI_2.5_ and LCI_5_ were both significantly higher in asthmatic patients as compared to non-asthmatic controls (Fig. 1A, p < 0.001). Likewise, S_acin_ and S_cond_ were both elevated and significantly different (Fig. 1B, p < 0.001 and p < 0.01). CV was 3.4% for LCI_2.5_ and 3.5% for LCI_5_ in patients with asthma. No significant differences were found as compared to 3.0% for LCI_2.5_ (p = 0.28) and 3.2% for LCI_5_ (p = 0.37) in non-asthmatic controls. Parameters of local ventilation heterogeneity showed considerably larger variation. CVs were 42% for S_acin_ and 82% for S_cond_ in asthma as compared to 39% (p = 0.74) and 127% (p = 0.17) in non-asthmatic controls, respectively. ΔFRC_pleth-MBW_ was 0.04 ± 0.52 L in non-asthmatic controls as compared to 0.61 ± 0.75 L in asthmatic patients (Fig. 1C, p<<0.001). Asthmatic patients with normal spirometry also had an elevated ΔFRC_pleth-MBW_ of 0.46 ± 0.62 as compared to non-asthmatic controls (p < 0.001).

**Table 2:** Lung function testing (whole cohort).

	unit	asthma (n = 91)		non-asthmatic controls (n = 47)		p-value
		value	range	value	range	
***spirometry***						
FEV_1_/VC	%pred	84 ± 14	42–112	99 ± 7	84–115	<<0.001*
FEV_1_/FVC	%	72 ± 10	44–92	83 ± 6	71–99	<<0.001*
FEV_1_	%pred	80 ± 20	22–132	101 ± 13	65–135	<<0.001*
VC	%pred	95 ± 18	42–132	102 ± 13	64–124	0.01*
MEF_75_	%pred	61 ± 27	11–122	96 ± 25	48–162	<<0.001*
MEF_50_	%pred	48 ± 26	10–127	88 ± 27	35–170	<<0.001*
MEF_25_	%pred	36 ± 23	9–107	67 ± 28	13–141	<<0.001*
***body plethysmography***						
TLC	%pred	109 ± 18	65–153	108 ± 11	87–128	0.60
RV	%pred	143 ± 51	48–451	125 ± 27	79–201	<0.01*
RV/TLC	%	45 ± 11	22–80	37 ± 8	21–57	<<0.001*
FRC_pleth_	L	3.4 ± 0.9	1.1–6.0	3.1 ± 0.5	2.3–4.2	0.05
TLCO/VA	%pred	88 ± 15	62–122	96 ± 10	80–116	<0.001*
TLCO	%pred	79 ± 16	41–125	87 ± 10	66–108	<0.001*
ΔTLCO	%pt	12 ± 9	0–44	11 ± 8	0–33	0.50
***impulse oscillometry***						
D5–20	%	40 ± 41	0–265	14 ± 11	0–47	<0.001*
Ax	—	1.5 ± 1.8	0.0–10.8	0.3 ± 0.2	0.0–1.1	<0.001*
F_res_	Hz	19 ± 8	6–44	11 ± 4	3–22	<0.001*
***multiple breath wash-out***						
LCI_2.5_	—	8.6 ± 1.8	5.5–13.6	7.0 ± 0.9	5.7–8.9	<0.001*
LCI_5_	—	6.6 ± 1.3	4.5–10.4	5.6 ± 0.6	4.5–7.0	<0.001*
FRC_MBW_	L	2.7 ± 0.9	1.0–6.1	3.1 ± 0.8	1.8–4.6	0.01*
S_acin_	L^−1^	0.17 ± 0.15	−0.2-0.65	0.07 ± 0.09	−0.35-0.21	<0.001*
S_cond_	L^−1^	0.06 ± 0.04	−0.03-0.18	0.04 ± 0.04	−0.07-0.13	<0.01*

FEV_1_: forced expiratory volume in one second, (F)VC: (forced) vital capacity, MEF: maximum expiratory flow at 75, 50 and 25% of FVC, TLC: total lung capacity, RV: residual volume, TLCO(/VA): transfer factor (corrected for ventilated alveolar volume), FRC: functional residual capacity, D5–20: frequency dependence of resistance, AX: area under reactance curve, Fres: resonance frequency, LCI: lung clearance index at 2.5% and 5% stopping points, S_acin_: acinar ventilation heterogeneity, S_cond_: conductive ventilation heterogeneity. %pred: percent of predicted, %pt: percentage points, *statistically significant p < 0.05.

**Figure 2:**
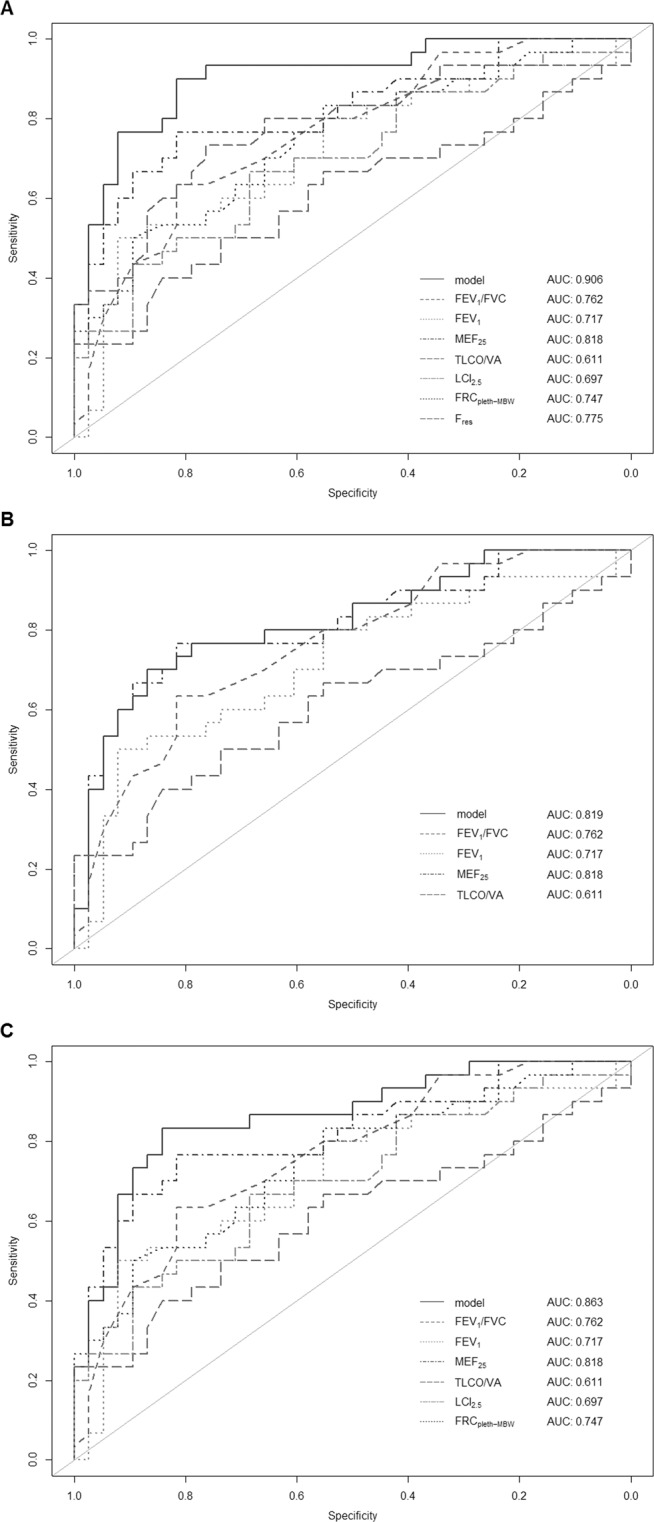
Diagnostic performance. ROC analysis for differentiation of asthmatic patients with normal conventional lung function testing (n = 41, FEV_1_ ≥ 80% predicted, FEV_1_/FVC ≥ 70%) and non-asthmatic controls (n = 47). (**A**) Good diagnostic performance (AUC = 0.906) of the model when including predictors identified during regularization. (**B**) Significantly lower accuracy (AUC = 0.819, p < 0.05) when including only parameters of conventional lung function testing. (**C**) Addition of MBW without IOS parameters also improves diagnostic performance (AUC = 0.863, p = 0.2).

In the regularized generalized linear model used for variable selection, FEV_1_/FVC, maximum expiratory flow at 25% of FVC (MEF_25_), LCI_2.5_, FRC_MBW_, area under the reactance curve (Ax), resonance frequency (F_res_), TLCO/VA and ΔFRC_pleth-MBW_ remained as predictors for asthma after adjustment for age and smoking status. ROC analysis revealed an AUC of 0.906 for the differentiation from non-asthmatic controls that exceeded diagnostic performance of the individual parameters (Fig. 2A). Diagnostic accuracy was significantly lower when only including parameters of conventional lung function testing (AUC = 0.819, p < 0.05, Fig. 2B). A model only consisting of conventional lung function testing and MBW but without IOS parameters yielded an improved AUC of 0.863 (p = 0.2, Fig. 2C). No differences were found between LCI_5_ (AUC = 0.683) and LCI_2.5_ (AUC = 0.697, p = 0.27) when used as distinct parameters. S_acin_ and S_cond_ had lower diagnostic accuracy (AUC = 0.615 and 0.593).

Mean total wash-out time was 102 ± 37 s in non-asthmatic controls and 102 ± 39 s in patients with asthma for LCI_2.5_. When using the earlier LCI_5_ stopping point, wash-out times were significantly reduced by 24 s (p<<0.001) and 28 s (p<<0.001), respectively.

## Discussion

We were able to demonstrate that MBW measurements are feasible in patients with bronchial asthma. Global and local ventilation heterogeneity is increased as compared to non-asthmatic controls. These differences persist when comparing asthmatic patients with normal conventional lung function parameters to non-asthmatic controls. MBW parameters therefore may contain additional information that can be used to identify asthmatic patients with abnormal ventilation missed by current tests. Including parameters of ventilation heterogeneity and small airway disease significantly improves diagnostic accuracy of our prediction model. Using an earlier stopping point does not affect diagnostic performance of LCI while considerably reducing wash-out duration.

Our findings are in accordance with previously published data in asthmatic children. Residual global ventilation heterogeneity could be identified despite well-controlled disease and normal FEV_1_. We also found significant differences between adults with asthma and non-asthmatic in acinar but not conductive ventilation heterogeneity. In contrast, parameters of regional inhomogeneity did not differ significantly between asthmatic and healthy children^25^. LCI was moderately, but statistically significantly elevated in a smaller cohort of asthmatic children while effect of acute bronchodilator therapy with salbutamol was detected. However, a very small subgroup of four patients with large improvement of airway obstruction were identified to show a decreased ventilation heterogeneity^41^. MBW indices were found not to be ideal for assessing bronchodilator reversibility in younger children with stable wheeze^42^. Although measurements using our setup themselves were demonstrated to be rather time effective, an additional interval should have been introduced after the forced breathing manoeuvres required during spirometry and plethysmography. We therefore decided to focus on the pre-bronchodilator in our investigation acknowledging the potential value of additional post-bronchodilator measurements to be further evaluated in adults. Abnormalities identified visually using magnetic resonance imaging (MRI) were shown to significantly correspond to poor asthma control^43^. In adult respiratory care, application of MBW comprises numerous potential scenarios. Kjellberg and co-workers were able to identify a “small airway subtype” in asthma patients. It was linked to reduced FEV_1_, smoking and blood eosinophilia and may potentially improve phenotyping. IOS and N_2_-MBW were found to give complementary information about peripheral airway dysfunction. These are attributable to different domains of small airway disease addressed by either test as recently demonstrated^5^. Altered values of D5–20 and LCI were found in 23% and 45% of asthmatic patients with normal FEV_1_^44^. This favourably corresponds to our findings at hand in asthma and also with previous reports in COPD using IOS and MBW, respectively. Small airway disease was detected with IOS even in early stages and linked to typical symptoms such as dyspnoea^45^. In early stages, IOS parameters outperformed conventional spirometry when differentiating heavy smokers with normal lung function as well as early-stage COPD^46^. Likewise, LCI was shown to be elevated in COPD in general and increases with GOLD stage^20^. Even in absence of spirometric obstruction, LCI was already pathologically altered^22^. Differences in FRC as measured by plethysmography and MBW were described previously in patients with obstructive lung disease^39,47,48^. When using an exogenous tracer gas such as SF_6_, these can be easily explained as only ventilated areas will contribute to FRC determination. In contrast, all compressible gas volumes are measured in body plethysmography^49^. Correspondingly, we found an increased ΔFRC_pleth-MBW_ in asthmatic patients even in absence of spirometric changes. Inclusion of this MBW parameter increases diagnostic accuracy already considerably. A model based on all objectively selected parameters including MBW and IOS further improves diagnostic accuracy. This outperforms both conventional tests and as well as distinct parameters from novel tests alone. Therefore, ΔFRC_pleth-MBW_ itself may yield diagnostic information due to tracer gas not reaching non-ventilated areas when obstruction is present. Taken together, evidence exists that novel parameters of small airway disease improve diagnostic accuracy and have therapeutic implications.

In clinical and scientific routine, several metrological aspects should be considered. Recently, it was demonstrated that duplicate LCI measurements are sufficient in adults^39^ and preschool children^50^. Intra-test repeatability is not affected while total test time is reduced significantly. These findings could be replicated in pre-school children suffering from cystic fibrosis using an N_2_-based setup^51^ with all three studies opposing current general recommendations^35^. Although we were able to acquire at least two successful runs in at least 90% of asthmatic patients and all non-asthmatic controls, using an earlier stopping point may eventually contribute to even higher success rates. In accordance with our findings, LCI_5_ was shown to be reliable in childhood and adolescence^52^ with equal diagnostic performance to the traditional stopping point^53^. However, this approach requires separate normal values that may be overcome using predictive modelling. An algorithm was demonstrated to reliably extrapolate LCI_2.5_ from earlier stopping points with reasonable accuracy. Additionally, this has the potential to further reduce wash-out time by up to 41% when using a 10% cut-off for prediction^54^. A similar 43% reduction in wash-out time can be potentially achieved when estimating LCI using Bayesian modelling^55^. Meaningful differences in MBW outcome measures depending on the test gases have been described^20,56^. In general, nitrogen-based systems reproducibly yield higher absolute LCI readings than SF_6_-MBW^49,57,58^. N_2_ has a higher diffusion rate and smaller molar mass as compared to SF_6_ resulting in a more proximal diffusion-convection front^35^. While SF_6_ may not reach very poorly ventilated regions during wash-in, endogenous N_2_ may prolong wash-out from these regions resulting in higher LCI values. Hence, attention has to be paid to test gas choice when interpreting MBW results^59^. Despite the above-mentioned potential advantages of SF_6_ -MBW, it should be noted that tracer gas still is associated with considerable costs. The system we used is commercially available and has regulatory approval (FDA and CE mark). Nevertheless, SF_6_ is not allowed for medical use in several countries. While no solid reference values are available for closed-circuit SF_6_-MBW in adults to date, age was identified the major factor influencing factor for nitrogen-based LCI measurements in adults and therefore prespecified as a control variable in our model^60^. The influence of height seems to be negligible in patients older than six years while a nonlinear decrease pattern was described when using SF_6_ as tracer gas^61^. Single breath N_2_-wash-out can be seen as a potentially time efficient alternative to MBW testing. However, sensitivity was found to be lower in adult asthmatic patients as compared to MBW and may therefore be a marker of more severe disease^62^. Within-test repeatability found in our study is comparable to previously published data in stable adults showing CVs between 2.5 and 4.5% for LCI using SF_6_ as tracer gas^37,39,63,64^. When paediatric populations are investigated, CV tends to be slightly higher ranging from 3.2 to 8.5%^50,54,65^. Likewise, considerably larger CVs have been shown for S_cond_ and S_acin_ with more variability in non-asthmatic controls than in COPD ranging from 17 to 73%^66^. This corresponds favourably to our findings. We found similar CV ranges in both groups except for S_cond_ in non-asthmatic controls showing a higher repeatability of 127%. We can reasonably exclude poor quality control as all lung function tests were independently assessed by two experienced investigators. Rather this may be attributable to the overall lower values of S_cond_ making it more vulnerable to variation in non-asthmatic controls. The fact that variation was largest in non-asthmatic controls lets us conclude that this does not negatively affect diagnostic performance in disease. In summary, our findings add well to previous reports in paediatric cohorts and different test gases and may further facilitate the routine use of MBW. Results obtained with different tracer gases should not be compared directly.

When interpreting our results, several points should be taken into consideration. First, we did not obtain structured information on asthma control, symptoms or medication. Moreover, diagnosis relied on a respiratory specialist evaluation according to Global Initiative for Asthma (GINA) criteria^1^. Although this clinical information would have been valuable, we do not consider this a major drawback of our study as we primarily set out for feasibility evaluation. The same holds for biomarkers such as laboratory parameters or FeNO. The latter did not show differences between asthmatic children and controls whereas LCI was significantly elevated^25^. Second, our cross-sectional design does not allow to provide estimates of the minimal clinical important difference for SF_6_-MBW. Due to the high intra-test repeatability, it should be possible to detect small longitudinal changes while further research is required. Nevertheless, our findings contribute to closing another knowledge gap in adult MBW testing. Personalized medicine cannot be reduced to a single biomarker or functional test. Assessment of ventilation heterogeneity using MBW may be one brick in the wall for improving phenotyping of asthmatic patients that warrants further investigation. Third, reference values were derived from the revised 1993 version of European Community for Steel and Coal (ECSC) equations^38^. This was mainly as the current Global Lung Function Initiative (GLI) reference values are restricted to spirometry. Requiring separate reference calculations for whole-body plethysmography, we aimed to avoid any mixing of the two statistically differing concepts.

## Conclusions

SF_6_-MBW measurements are feasible in adult patients with bronchial asthma. Indices of global (LCI) and local ventilation heterogeneity (S_acin_, S_cond_) both are elevated as compared to non-asthmatic controls. Differences persist in asthmatic patients without obstruction in spirometry. MBW parameters therefore may contain additional information that can be used to identify asthmatic patients with abnormal ventilation missed by current tests. For LCI as the most important MBW outcome measure, using an earlier stopping point does not affect diagnostic performance while considerably reducing the duration of the wash-out procedure. Reproducibility was high for both LCI stopping points. S_acin_ and S_cond_ both showed larger variability.

## Supplementary information

Supplementary Information.

## Data Availability

The datasets generated during and analysed during the current study are available from the corresponding author on reasonable request.

## References

[1] Global Initiative for Asthma. Global Strategy for Asthma Management and Prevention. Available from: www.ginaasthma.org

[2] Partridge MR, van der Molen T, Myrseth SE, Busse WW (2006). Attitudes and actions of asthma patients on regular maintenance therapy: the INSPIRE study. BMC Pulm. Med..

[3] Larsson K (2018). Prevalence and management of severe asthma in primary care: an observational cohort study in Sweden (PACEHR). Respir. Res..

[4] Rabe KF, Vermeire PA, Soriano JB, Maier WC (2000). Clinical management of asthma in 1999: the Asthma Insights and Reality in Europe (AIRE) study. Eur. Respir. J..

[5] Postma DS (2019). Exploring the relevance and extent of small airways dysfunction in asthma (ATLANTIS): baseline data from a prospective cohort study. Lancet Respir. Med..

[6] Carr TF, Altisheh R, Zitt M (2017). Small airways disease and severe asthma. World Allergy. Organ. J..

[7] Kuyper LM (2003). Characterization of airway plugging in fatal asthma. Am. J. Med..

[8] de Magalhaes Simoes S (2005). Inflammatory cell mapping of the respiratory tract in fatal asthma. Clin. Exp. Allergy.

[9] Carroll N, Cooke C, James A (1997). The distribution of eosinophils and lymphocytes in the large and small airways of asthmatics. Eur. Respir. J..

[10] de Medeiros Matsushita M (2005). Airway proteoglycans are differentially altered in fatal asthma. J. Pathol..

[11] Foy BH (2019). Lung Computational Models and the Role of the Small Airways in Asthma. Am. J. Respir. Crit. Care Med..

[12] Shi Y (2012). Relating small airways to asthma control by using impulse oscillometry in children. J. Allergy Clin. Immunol..

[13] Takeda T (2010). Relationship between small airway function and health status, dyspnea and disease control in asthma. Respiration.

[14] Nair A, Ward J, Lipworth BJ (2011). Comparison of bronchodilator response in patients with asthma and healthy subjects using spirometry and oscillometry. Ann. Allergy Asthma Immunol..

[15] Garcia-Quero, C. *et al*. Small Airway Dysfunction Impairs Quality of Life Among Smokers With No Airflow Limitation. *Arch Bronconeumol* (2019). 10.1016/j.arbres.2019.01.006 30824207

[16] Sugawara H, Saito A, Yokoyama S, Tsunematsu K, Takahashi H (2019). Comparison of therapeutic effects of inhaled corticosteroids on three subtypes of cough variant asthma as classified by the impulse oscillometry system. Respir. Res..

[17] Buhl, R. *et al*. Severe eosinophilic asthma: a roadmap to consensus. *Eur Respir J* **49**, (2017). 10.1183/13993003.00634-2017 28461308

[18] Korevaar DA (2015). Diagnostic accuracy of minimally invasive markers for detection of airway eosinophilia in asthma: a systematic review and meta-analysis. Lancet Respir. Med..

[19] Price DB (2018). Fractional exhaled nitric oxide as a predictor of response to inhaled corticosteroids in patients with non-specific respiratory symptoms and insignificant bronchodilator reversibility: a randomised controlled trial. Lancet Respir. Med..

[20] Bell AS, Lawrence PJ, Singh D, Horsley A (2018). Feasibility and challenges of using multiple breath washout in COPD. Int. J. Chron. Obstruct Pulmon Dis..

[21] Fähndrich S (2016). Lung Clearance Index is Increased in Patients with COPD - LCI Measurements in the Daily Routine. J. Pulm. Respir. Med. 2016.

[22] Trinkmann F (2016). Multiple breath washout (MBW) using sulfur hexafluoride – Proof of concept in COPD. Eur. Respir. J..

[23] Trinkmann, F. *et al*. Small Airway Disease in Pulmonary Hypertension-Additional Diagnostic Value of Multiple Breath Washout and Impulse Oscillometry. *J Clin Med* **7** (2018). 10.3390/jcm7120532 PMC6306708 30544842

[24] Gustafsson PM (2007). Peripheral airway involvement in CF and asthma compared by inert gas washout. Pediatr. Pulmonol..

[25] Macleod KA (2009). Ventilation heterogeneity in children with well controlled asthma with normal spirometry indicates residual airways disease. Thorax.

[26] Verbanck S, Schuermans D, Paiva M, Vincken W (2003). Nonreversible conductive airway ventilation heterogeneity in mild asthma. J. Appl. Physiol..

[27] Farah CS (2012). Ventilation heterogeneity predicts asthma control in adults following inhaled corticosteroid dose titration. J. Allergy Clin. Immunol..

[28] Thompson BR (2013). Peripheral lung function in patients with stable and unstable asthma. J. Allergy Clin. Immunol..

[29] Farah CS (2012). The role of the small airways in the clinical expression of asthma in adults. J. Allergy Clin. Immunol..

[30] Hardaker KM (2013). Ventilation heterogeneity is associated with airway responsiveness in asthma but not COPD. Respir. Physiol. Neurobiol..

[31] Kane M (2017). Correcting for tissue nitrogen excretion in multiple breath washout measurements. PLoS One.

[32] Lenherr, N. *et al*. Leaks during multiple-breath washout: characterisation and influence on outcomes. *ERJ Open Res* **4** (2018). 10.1183/23120541.00012-2017 PMC5827412 29497618

[33] Sullivan, L., Forno, E., Pedersen, K., Nielsen, J. G. & Weiner, D. J. Nitrogen back-diffusion during multiple-breath washout with 100% oxygen. *Eur Respir J* **50** (2017). 10.1183/13993003.00679-2017 28889111

[34] Nielsen JG (2014). Lung clearance index: should we really go back to nitrogen washout?. Eur. Respir. J..

[35] Robinson PD (2013). Consensus statement for inert gas washout measurement using multiple- and single- breath tests. Eur. Respir. J..

[36] Clemensen P, Christensen P, Norsk P, Gronlund J (1994). A modified photo- and magnetoacoustic multigas analyzer applied in gas exchange measurements. J. Appl. Physiol..

[37] Horsley, A. R. *et al*. Closed circuit rebreathing to achieve inert gas wash-in for multiple breath wash-out. *ERJ Open Res* **2** (2016). 10.1183/23120541.00042-2015 PMC5005150 27730167

[38] Quanjer PH (1993). Lung volumes and forced ventilatory flows. Report Working Party Standardization of Lung Function Tests, European Community for Steel and Coal. Official Statement of the European Respiratory Society. Eur. Respir. J. Suppl..

[39] Trinkmann F (2017). Multiple breath washout testing in adults with pulmonary disease and healthy controls - can fewer measurements eventually be more?. BMC Pulm. Med..

[40] R Core Team. R: A Language and Environment for Statistical Computing. R Foundation for Statistical Computing, Vienna, Austria. https://www.R-project.org/ (2017).

[41] Zwitserloot A, Fuchs SI, Muller C, Bisdorf K, Gappa M (2014). Clinical application of inert gas Multiple Breath Washout in children and adolescents with asthma. Respir. Med..

[42] Sonnappa S (2013). Repeatability and bronchodilator reversibility of lung function in young children. Eur. Respir. J..

[43] Svenningsen S, Nair P, Guo F, McCormack DG, Parraga G (2016). Is ventilation heterogeneity related to asthma control?. Eur. Respir. J..

[44] Kjellberg S, Houltz BK, Zetterstrom O, Robinson PD, Gustafsson PM (2016). Clinical characteristics of adult asthma associated with small airway dysfunction. Respir. Med..

[45] Crisafulli E (2017). Prevalence of Small-Airway Dysfunction among COPD Patients with Different GOLD Stages and Its Role in the Impact of Disease. Respiration.

[46] Su ZQ (2018). Significances of spirometry and impulse oscillometry for detecting small airway disorders assessed with endobronchial optical coherence tomography in COPD. Int. J. Chron. Obstruct Pulmon Dis..

[47] Gonem S (2014). Validation of a photoacoustic gas analyser for the measurement of functional residual capacity using multiple-breath inert gas washout. Respiration.

[48] Horsley A, Macleod K, Gupta R, Goddard N, Bell N (2014). Enhanced photoacoustic gas analyser response time and impact on accuracy at fast ventilation rates during multiple breath washout. PLoS One.

[49] Jensen R (2013). Multiple breath nitrogen washout: a feasible alternative to mass spectrometry. PLoS One.

[50] Robinson PD, Stocks J, Aurora P, Lum S (2013). Abbreviated multi-breath washout for calculation of lung clearance index. Pediatr. Pulmonol..

[51] Foong, R. E. *et al*. The clinical utility of lung clearance index in early cystic fibrosis lung disease is not impacted by the number of multiple-breath washout trials. *ERJ Open Res* **4** (2018). 10.1183/23120541.00094-2017 PMC5912932 29707562

[52] Yammine S, Singer F, Abbas C, Roos M, Latzin P (2013). Multiple-breath washout measurements can be significantly shortened in children. Thorax.

[53] Hannon D (2014). Shortened Lung Clearance Index is a repeatable and sensitive test in children and adults with cystic fibrosis. BMJ Open. Respir. Res..

[54] Gronbaek J (2016). New time-saving predictor algorithm for multiple breath washout in adolescents. Pediatr. Res..

[55] Mahar RK (2018). Bayesian modelling of lung function data from multiple-breath washout tests. Stat. Med..

[56] Yammine S, Lenherr N, Nyilas S, Singer F, Latzin P (2015). Using the same cut-off for sulfur hexafluoride and nitrogen multiple-breath washout may not be appropriate. J. Appl. Physiol..

[57] Nielsen N, Nielsen JG, Horsley AR (2013). Evaluation of the impact of alveolar nitrogen excretion on indices derived from multiple breath nitrogen washout. PLoS One.

[58] Guglani, L. *et al*. Difference between SF_6_ and N_2_ Multiple Breath Washout kinetics is due to N_2_ back diffusion and error in N_2_ offset. *J Appl Physiol* (1985) (2018). 10.1152/japplphysiol.00326.2018 30048204

[59] Bayfield, K. J. *et al*. Simultaneous sulfur hexafluoride and nitrogen multiple-breath washout (MBW) to examine inherent differences in MBW outcomes. *ERJ Open Research* **5** (2019). 10.1183/23120541.00234-2018 PMC6826248 31720295

[60] Verbanck S (2012). Ventilation heterogeneity in the acinar and conductive zones of the normal ageing lung. Thorax.

[61] Lum S (2013). Age and height dependence of lung clearance index and functional residual capacity. Eur. Respir. J..

[62] Kjellberg, S. *et al*. Utility of single versus multiple breath washout in adult asthma. *Clin Physiol Funct Imaging* (2018). 10.1111/cpf.12503 29368419

[63] Horsley AR (2008). Lung clearance index is a sensitive, repeatable and practical measure of airways disease in adults with cystic fibrosis. Thorax.

[64] Grillo L (2015). The reproducibility and responsiveness of the lung clearance index in bronchiectasis. Eur. Respir. J..

[65] Aurora P (2004). Multiple breath inert gas washout as a measure of ventilation distribution in children with cystic fibrosis. Thorax.

[66] Husemann K (2014). Double tracer gas single-breath washout: reproducibility in healthy subjects and COPD. Eur. Respir. J..

