# Supplementary material for: Feasibility and clinical applications of multiple breath wash-out (MBW) testing using sulphur hexafluoride in adults with bronchial asthma
Source: Sci Rep. 2020 Jan 30;10:1527. doi: 10.1038/s41598-020-58538-x (PMC6992773; doi:10.1038/s41598-020-58538-x)
Supplement: Supplementary file 1 — Supplementary Information. [file 41598_2020_58538_MOESM1_ESM.docx]

**Feasibility and clinical applications of multiple breath wash-out (MBW) testing using sulphur hexafluoride in adults with bronchial asthma**

Frederik Trinkmann, Steffi A. Lenz, Julia Schäfer, Joshua Gawlitza, Michele Schroeter, Tobias Gradinger, Ibrahim Akin, Martin Borggrefe, Thomas Ganslandt, Joachim Saur

**Online supplement**

**Classification criteria**

- **Non-asthmatic** **controls**
  - no history of pulmonary disease
  - absence of dyspnoea, cough, thoracic pain
  - self-reported smoking history < 10 pack years
  - normal lung function testing
    - normal shape of flow-volume curve
    - normal shape of flow-pressure curve
    - FEV_1_/VC >80%, TLC > 80%, TLCO/VA > 80% (all % of predicted)
- **Bronchial asthma**
  - clinical history and/or specialist diagnosis of bronchial asthma
  - respiratory symptoms compatible with asthma varying over time (wheeze, dyspnoea, chest tightness, cough)
  - variable and/or reversible obstructive ventilation disorder and/or airway hyperresponsiveness
  - exclusion of alternative explanation

**Acceptability Criteria for MBW**

- breathing pattern
  - stable tidal volume, no hyperventilation, no hypoventilation
  - no coughing
  - assessed clinically and in-silico
- variability
  - within 10% from the median FRC / LCI across triplicate tests
  - careful examination for technical issues if larger, but within 25%
  - rejection if larger > 25%
- no leak
  - equilibration between inspiratory and expiratory SF_6_ concentrations during wash-in
  - no sudden drop in inspiratory SF_6_ concentration during wash-in
  - check of volume curve during wash-out
- test termination
  - at least three consecutive breaths with end tidal SF_6_ concentrations <1/40th of starting value
- test conduction
  - no excessive swallowing (clinical assessment)
  - sufficient interval between tests

(twice the wash-out time or measurement of resituated gas concentrations)

| **Table S1 Reversibility testing (Asthma, n=63)** | | | | | | | | | | | | | | |  |  |
| --- | --- | --- | --- | --- | --- | --- | --- | --- | --- | --- | --- | --- | --- | --- | --- | --- |
|  | | |  | | **absolute change** | | |  | **positive rate** | | | |  |  | |  |
|  | | | **unit** | | **value** | **SD** | |  | **n** | **%** | | |  |  | |  |
| FEV_1_ | | | % | | 13 | ±12 | |  | 24 | 38 | | |  |  | |  |
| RV |  | | % | | 9 | ±20 | |  | 13 | 21 | | |  |  | |  |
| D5-20 | | | % | | 24 | ±51 | |  | 18 | 29 | | |  |  | |  |
| Ax | | | % | | 34 | ±46 | |  | 24 | 38 | | |  |  | |  |
| FEV_1_: >12% and 200 ml increase according to ^1^, RV: >20% decrease according to ^2^, D5-20 >50%, and AX: >50% decrease according to ^3^. Other abbreviations are identical with Table 2. | | | | | | | | | | | | | | |  |  |
|  | |  | |  | |  |  | | | |  |  | | |  |  |

| **Table S2 Lung function testing (asthmatic patients with normal spirometry)** | | | | | | | | | |
| --- | --- | --- | --- | --- | --- | --- | --- | --- | --- |
|  |  | **Asthma (n=41)** | |  | **non-asthmatic**  **controls (n=47)** | |  |  |  |
|  | **unit** | **value** | **range** |  | **value** | **range** |  |  | **p-value** |
| *spirometry* | |  |  |  |  |  |  |  |  |
| FEV_1_/VC | %pred | 93±9 | 79-112 |  | 99±7 | 84-115 |  |  | <0.001* |
| FEV_1_/FVC | % | 79±5 | 70-87 |  | 83±6 | 71-99 |  |  | <0.001* |
| FEV_1_ | %pred | 95±12 | 81-132 |  | 101±13 | 65-135 |  |  | <0.001* |
| VC | %pred | 104±11 | 74-132 |  | 102±13 | 64-124 |  |  | 0.73 |
| MEF_75_ | %pred | 84±19 | 53-122 |  | 96±25 | 48-162 |  |  | <0.01* |
| MEF_50_ | %pred | 68±23 | 32-127 |  | 88±27 | 35-170 |  |  | <0.001* |
| MEF_25_ | %pred | 47±23 | 13-107 |  | 67±28 | 13-141 |  |  | <0.001* |
| *body plethysmography* | | |  |  |  |  |  |  |  |
| TLC | %pred | 109±15 | 73-146 |  | 108±11 | 87-128 |  |  | 0.65 |
| RV | %pred | 129±30 | 48-208 |  | 125±27 | 79-201 |  |  | 0.45 |
| RV/TLC | % | 40±8 | 22-57 |  | 37±8 | 21-57 |  |  | 0.05 |
| FRC_pleth_ | L | 3.1±0.8 | 1.1-4.7 |  | 3.1±0.5 | 2.3-4.2 |  |  | 0.95 |
| *gas transfer* | | | | | | | | | |
| TLCO/VA | %pred | 87±15 | 63-122 |  | 96±10 | 80-116 |  |  | <0.01* |
| TLCO | %pred | 82±15 | 50-125 |  | 87±10 | 66-108 |  |  | 0.07 |
| ΔTLCO | %pt | 8±5 | 0-20 |  | 11±8 | 0-33 |  |  | 0.14 |
| *impulse oscillometry* | |  |  |  |  |  |  |  |  |
| D5-20 | % | 27±27 | 0-130 |  | 14±11 | 0-47 |  |  | <0.01* |
| Ax | - | 0.9±1.1 | 0.0-5.0 |  | 0.3±0.2 | 0.0-1.1 |  |  | <0.01* |
| F_res_ | Hz | 16±7 | 6-36 |  | 11±4 | 3-22 |  |  | <<0.001* |
| *multiple breath wash-out* | |  |  |  |  |  |  |  |  |
| LCI_2.5_ | - | 7.9±1.4 | 6-12 |  | 7.0±0.9 | 5.7-8.9 |  |  | <0.001* |
| LCI_5_ | - | 6.2±1.1 | 4.5-7.0 |  | 5.6±0.6 | 4.5-7.0 |  |  | <0.01* |
| FRC_MBW_ | L | 2.6±0.7 | 1.2-4.4 |  | 3.1±0.8 | 1.8-4.6 |  |  | <0.01* |
| S_acin_ | L^-1^ | .12±.14 | -.2-.65 |  | .07±.09 | -.35-.21 |  |  | 0.05 |
| S_cond_ | L^-1^ | .05±.04 | -.03-.17 |  | .04±.04 | -.07-.13 |  |  | 0.22 |
| FEV_1_: forced expiratory volume in one second, (F)VC: (forced) vital capacity, MEF: maximum expiratory flow at 75, 50 and 25% of FVC, TLC: total lung capacity, RV: residual volume, TLCO(/VA): transfer factor (corrected for ventilated alveolar volume), FRC: functional residual capacity, D5-20: frequency dependence of resistance, AX: area under reactance curve, F_res_: resonance frequency, LCI: lung clearance index at 2.5% and 5% stopping points, S_acin_: acinar ventilation heterogeneity, S_cond_: conductive ventilation heterogeneity. %pred: percent of predicted, %pt: percentage points, *statistically significant p<0.05. | | | | | | | | | |

1 Pellegrino, R. *et al.* Interpretative strategies for lung function tests. *Eur Respir J* **26**, 948-968, doi:10.1183/09031936.05.00035205 (2005).

2 McCartney, C. T., Weis, M. N., Ruppel, G. L. & Nayak, R. P. Residual Volume and Total Lung Capacity to Assess Reversibility in Obstructive Lung Disease. *Respir Care* **61**, 1505-1512, doi:10.4187/respcare.04323 (2016).

3 Smith, H., Reinhold, P. & Goldman, M. in *Lung Function testing: European Respiratory Society Monograph* Vol. 31 (eds R. Gosselink & H. Stam) 72-105 (European Respiratory Society, 2005).
